# *Clostridium perfringens* in the Intestine: Innocent Bystander or Serious Threat?

**DOI:** 10.3390/microorganisms12081610

**Published:** 2024-08-07

**Authors:** Xuli Ba, Youshun Jin, Xuan Ning, Yidan Gao, Wei Li, Yunhui Li, Yihan Wang, Jizhang Zhou

**Affiliations:** 1 State Key Laboratory for Animal Disease Control and Prevention, College of Veterinary Medicine, Lanzhou University, Lanzhou Veterinary Research Institute, Chinese Academy of Agricultural Sciences, Lanzhou 730000, China; 220220909200@lzu.edu.cn (X.B.); jinysh19@lzu.edu.cn (Y.J.); ningxuan0217@163.com (X.N.); ljp552119359@126.com (W.L.); 2 Gansu Province Research Center for Basic Disciplines of Pathogen Biology, Lanzhou 730046, China; liyunhuihou@163.com (Y.L.); wyihan233@163.com (Y.W.); 3 State Key Laboratory of Grassland Agro-Ecosystems, College of Pastoral Agriculture Science and Technology, Lanzhou University, Lanzhou 730020, China; gyd1357@163.com

**Keywords:** Zoonotic pathogen, *C. perfringens*, global epidemics, toxins

## Abstract

The *Clostridium perfringens* epidemic threatens biosecurity and causes significant economic losses. *C. perfringens* infections are linked to more than one hundred million cases of food poisoning annually, and 8–60% of susceptible animals are vulnerable to infection, resulting in an economic loss of more than 6 hundred million USD. The enzymes and toxins (>20 species) produced by *C. perfringens* play a role in intestinal colonization, immunological evasion, intestinal micro-ecosystem imbalance, and intestinal mucosal disruption, all influencing host health. In recent decades, there has been an increase in drug resistance in *C. perfringens* due to antibiotic misuse and bacterial evolution. At the same time, traditional control interventions have proven ineffective, highlighting the urgent need to develop and implement new strategies and approaches to improve intervention targeting. Therefore, an in-depth understanding of the spatial and temporal evolutionary characteristics, transmission routes, colonization dynamics, and pathogenic mechanisms of *C. perfringens* will aid in the development of optimal therapeutic strategies and vaccines for *C. perfringens* management. Here, we review the global epidemiology of *C. perfringens*, as well as the molecular features and roles of various virulence factors in *C. perfringens* pathogenicity. In addition, we emphasize measures to prevent and control this zoonotic disease to reduce the transmission and infection of *C. perfringens*.

## 1. Introduction

**Figure 1:**
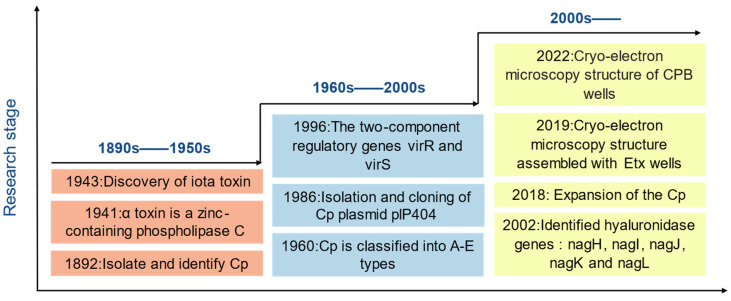
Roadmap of *C. perfringens.*

*Clostridium perfringens*, a Gram-positive bacillus, is ubiquitous in the environment as well as in the normal intestinal flora of humans and animals. *C. perfringens*, was first isolated and identified by William H. Welch in 1892 by necropsy (Figure 1), and is an intestinal conditionally pathogenic organism that becomes pathogenic when immunity is compromised, is an enteric conditionally pathogenic bacterium that becomes pathogenic when immunity is compromised. *C. perfringens*, with its rapid doubling speed and efficient resource utilization, coupled with its potent toxin production and environmental adaptability, can quickly colonize hosts and cause severe infections. These characteristics pose a serious threat to human health and global livestock (8–60% of susceptible animals are vulnerable to infection). Avian necrotizing enterocolitis causes a staggering global economic loss approximated at around 6 hundred million USD annually [1]. Antibiotic-associated diarrhea has been associated with a variety of bacterial infection factors, including *C. perfringens*, *Clostridium difficile*, *Staphylococcus aureus*, *Candida albicans*, and *Klebsiella oxytoca* [2]. Both *C. perfringens * and *Clostridioides difficile* can cause intestinal dysbiosis after antibiotic use. After the use of antibiotics such as β-lactams, β-lactamase inhibitors, and cephalosporins, *C. perfringens* produces enterotoxins, that cause antibiotic-associated diarrhea, in contrast, the use of antibiotics like fluoroquinolones, cephalosporins, and clindamycin, leads to *Clostridioides difficile* releasing toxins A and B, which also result in antibiotic-associated diarrhea [3]. New evidence suggests that antibiotic-associated diarrhea occurs in 5% to 30% of patients receiving antibiotics, with *C. perfringens* type A causing up to 15% of AAD cases and type F associated with 2% to 15% of AAD cases [3,4,5].

**Figure 2:**
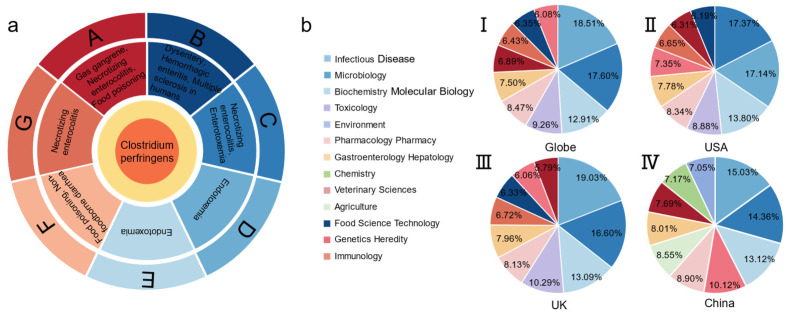
(**a**) Diseases caused by *C. perfringens.* (**b**) The direction of research on *C. perfringens* (Top 10): (**I**) Global (**II**) the United States (**III**) the United Kingdom (**IV**) China.

*C. perfringens* is classified into A–G strains and is known to cause various diseases in humans and animals, including gas gangrene, necrotizing enterocolitis, multiple sclerosis, antibiotic-associated diarrhea, and others (Figure 2). These pathogenic effects are linked to the production of exotoxins (e.g., α-toxin, β-toxin, enterotoxin, ε-toxin, ι-toxin, etc.) and enzymes (e.g., salivary lyase, phospholipase, hexosaminidase, among others) (Figure 2). Notably, *C. perfringens* infections cause a significant morbidity and lethality rate [6,7,8,9]. *C. perfringens* colonizes the intestinal tract, alters the intestinal environment, disrupts the intestinal microecological balance, and causes an inflammatory response, and its toxins attack multiple target organs, including the intestinal tract, kidneys, lungs, and heart, further damaging organs such as the kidneys, lungs, and heart [10]. In addition, *C. perfingens salivary lyase*, by promoting the adhesion and colonization of *C. perfringens* in the intestinal tract and the combination of toxins and intestinal cells, provides nutrition for *C. perfringens*, destroying the intestinal microecological balance and host immune system [11].

**Figure 3:**
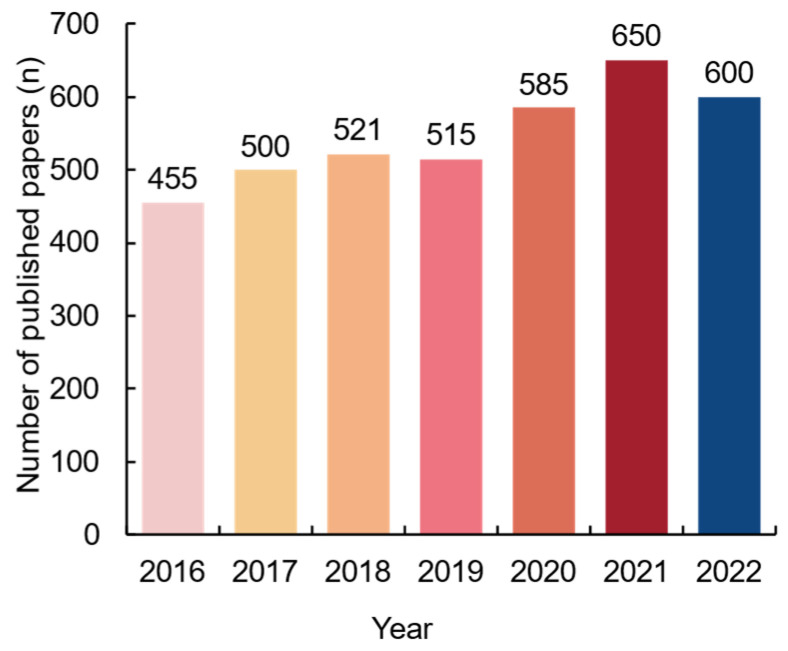
Literature published on *C. perfringens* in the past 7 years.

It is reasonable to assume that the primary treatment of *C. perfringens* disease is the precise elimination of *C. perfringens* colonization of the intestinal tract, with antibiotics now serving as the cornerstone. However, studies have demonstrated that prolonged antibiotic usage may disrupt the intestinal flora, decrease intestinal short-chain fatty acid concentrations, alter carbohydrate and bile acid uptake, and increase the pathogenicity of *C. perfringens* [12,13]. In addition, when the use of ionophore for the prevention and treatment of coccidiosis is discontinued, the bacterial flora in the intestinal tract of poultry may become unbalanced, leading to a rapid proliferation of *C. perfringens*, which can trigger dysbiosis. Meanwhile, *C. perfringens* acquire antibiotic resistance over time through genetic mutation, horizontal gene transfer, genetic resistance loading, and natural selection. Nearly 1.27 million individuals worldwide succumbed to antibiotic resistance in 2019, and global GDP and trade losses due to antibiotic resistance are projected to be as high as $8.5 billion and $2.3 billion, respectively, by 2025 [14,15]. Therefore, the solution to *C. perfringens* disease lies in the production of organic acids, enzymes, prebiotic oligosaccharides, and microecological agents at the multidisciplinary intersection of environment, chemistry, and medicine (Figure 2 and Figure 3).

Overall, this work examines the spatial and temporal evolutionary features of *C. perfringens*, as well as the pathogenic mechanisms of enzymes and major toxins produced by *C. perfringens*. Furthermore, we critically review the therapeutic perspectives of antibiotics, vaccines, and other biologics against *C. perfringens* to provide the most up-to-date research-based solutions to researchers, physicians, students, and the general.

### 1.1. C. perfringens Threatens Global Biological Public Safety

#### 1.1.1. Humans

Foodborne illness, bacteremia, and antibiotic-associated diarrhea (antibiotic-associated diarrhea, AAD) caused by *C. perfringens* are widespread worldwide, including the United Kingdom, the United States, Canada, and China, where there is a high prevalence of infection and cross-contamination through vectors such as raw meat, raw milk, and spices. Emerging evidence indicates that *C. perfringens* cause roughly 13% of foodborne food poisoning, 5–20% of AAD, and a portion of non-foodborne diarrhea [12,16] (Table 1). *C. perfringens*-related foodborne infections were predominantly reported in the Eastern region of the UK (2012–2014). These infections were particularly prevalent in communal settings, such as nursing homes. Alarmingly, this resulted in approximately 25 fatalities per year from *C. perfringens* [17,18]. Food poisoning caused by *C. perfringens* infection affects roughly 1 million individuals in the United States, about 900,000 people in England and approximately 16,000 people in Canada (2013) each year [19,20,21] (Table 1). Studies have shown that *C. difficile* is a major cause of ADD, and the incidence of *C. difficile* AAD is approximately four times higher than that of *C. perfringens* AAD [22]. In Iran, approximately 13.3% of patients diagnosed with antibiotic-associated diarrhea tested positive for *C. perfringens* infection, with type A being the most prevalent (Table 1). The prevalent type in central China (2018–2019) was F, accounting for 49.5% of cases [23,24]. *C. perfringens* is transmitted and cross-infected by various carriers, including raw meat and spices. For instance, the prevalence of *C. perfringens* in raw poultry products sold in different countries, including the United States, Japan, India, and Canada, varies significantly between samples, ranging from 6% to 97% [25]. The examination of raw meat samples in a slaughterhouse region in Shaanxi, China (2018–2019) revealed that 21.2% were isolated as type A *C. perfringens* (with 80.8% prevalence), and 19.2% as type D *C. perfringen* [26]. The detection rate in Kazakhstan was 28%, with a predominance of type A [27]. In China, cross-infection of *C. perfringens* during rearing and milking on dairy farms is present, with a prevalence of 22.3% [28]. *C. perfringens* spores can be found in spices in the United States, making them a vector for foodborne transmission of *C. perfringens* [29].

#### 1.1.2. Animal

*C. perfringens* may infect horses, sheep, goats, cattle, pigs, chickens, and other animals through their digestive tracts. *C. perfringens* type A is the most common, but types B, C, D, E, F, and G are also present in small amounts [31,32,33,34]. The prevalence of *C. perfringens* was roughly 8% of healthy horses in Switzerland (2013–2016) [35] (Table 2). The prevalence of enterotoxemia in sheep and goats was 45.2% in Samsun governorate, Turkey (2014–2015) [36]. The prevalence of *C. perfringens* was 17.5% lambs in Kalubiya and Menofia governorates, Egypt (2021–2022) [37] (Table 2). The prevalence of *C. perfringens* in India (2000–2021) was detected among different animal species as follows: pigs (60%) > sheep (56.3%) > goats (38.7%) > cattle (35%) [38] (Table 2). *C. perfringens* was detected in poultry farms in Weifang, Guangdong, Taian, and Pingyin at a remarkably high rate of 38.42% [36]. The isolation rate of *C. perfringens* in sheep in Gansu (2019–2020) was 14.7% (Table 2). Furthermore, in commercial farms in Anhui, Guangdong, Guangxi, and Fujian provinces (2020), the isolation rate of *C. perfringens* was notably higher, reaching 44.173% [39,40] (Table 2).

#### 1.1.3. Economy

*C. perfringens*, a globally widespread bacterium, can induce various illnesses with significant global economic consequences. *C. perfringens*-related food poisoning costs the United States economy roughly $ thirty billion, with type A accounting for approximately $500 million and type F accounting for approximately $310 million [41,42,43]. In the United Kingdom, the cost of treating necrotizing small bowel colitis in preterm infants caused by *C. perfringens* is approximately £130,000 per year [44]. Avian necrotizing enterocolitis causes a global economic loss of nearly $600 million each year [45].

## 2. Virulence Mechanisms

The pathogenic mechanism of *C. perfringens* includes intestinal colonization and proliferation, immune escape, toxin production, imbalance in the intestinal micro-ecosystem, disruption of the intestinal mucosa, and the formation of gas-producing pods, adversely impacting the host organism. Overall, the main pathogenic factors are multi-enzymes and toxins (α, β, ε, ι and others).

### 2.1. Enzymes

The intestinal mucus layer serves as a crucial physiological structure on the intestinal mucosa, acting as the primary line of defense for natural immunity. This barrier primarily comprises highly glycosylated mucins, which trap microorganisms, particulate matter, and other harmful substances, effectively protecting the intestinal mucosa from direct aggression. C. *perfringens*, an intestinal microorganism, can produce salivary lyase (NanH, NanI and NanJ, etc.), hexosaminidase, galactosidase, fucoidan, phospholipase, and other enzymes [46]. Different *C. perfringens* strains secrete different salivary lyase enzymes that remove terminal salivary acids from the intestinal mucus layer, resulting in mucin degradation [46]. In addition, *C. perfringens* potentially utilizes salivary acid as a carbon source. This metabolic trait facilitates the colonization of the intestinal tract by *C. perfringens* and enhances the toxic effects of its toxins on host cells [11,47].

### 2.2. Toxin

#### 2.2.1. Alpha Toxin

**Figure 4:**
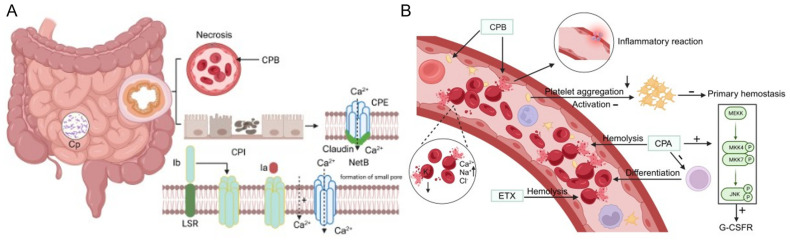
(**A**) Vascular pathogenesis of *C. perfringens* CPA: *C. perfringens* alpha toxin, CPB: *C. perfringens* beta toxin, ETX: Epsilon toxin, G-CSFR: granulocyte colony-stimulating factor receptor, MEKK: mitogen-activated protein kinase kinase kinase, MKK4: mitogen-activated protein kinase kinase 4, MKK7: mitogen-activated protein kinase kinase 7, JNK: c-Jun N-terminal kinase (**B**) Intestinal pathogenesis of *C. perfringens*. Cp: *C. perfringens*, CPB: *C. perfringens* beta toxin, CPE: *C. perfringens* enterotoxin, CPI: Iota toxin, NetB: Necrotizing Enterocolitis B-like toxin, LSR: lipolysis-stimulated lipoprotein receptor. + denotes promotion and − denotes inhibition.

*C. perfringens* alpha toxin (CPA) is a member of the phospholipase C family. The CPA has two structural domains: an N domain with nine tightly stacked α-helices and a C domain with eight antiparallel β-intercalated structures [48]. All types of *C. perfringens* secrete CPA. CPA inhibits granulopoiesis and erythroid differentiation while increasing vascular permeability and hemolysis [49,50]. CPA creates an anaerobic environment for *C. perfringens* growth by activating phosphatidylinositol turnover and thromboxane A2 synthesis, causing aortic constriction and reduced blood supply to the tissues [51,52]. Moreover, CPA inhibits erythropoiesis by blocking erythroid lineage differentiation, causing erythrocyte lysis and hemolysis, thereby providing an anaerobic environment for *C. perfringens* growth [53]. Furthermore, CPA activates the c-Jun N-terminal kinase (JNK) signaling pathway (Figure 4A). This stimulation increases the secretion of colony-stimulating factor (G-CSF). However, it concurrently suppresses the expression of colony-stimulating factor receptor (G-CSFR) (Figure 4A). This dual effect influences G-CSF-mediated granulocyte production and release, impacting the immune response and the ability of the body to fight infection [54]. More, CPA binds to the ganglioside receptor GM1a and is able to promote the degradation of sphingolipids and phosphatidylcholine in the cytoplasmic membrane, causing a pro-inflammatory response and exacerbating tissue infection aggravation [21].

#### 2.2.2. Beta Toxin

Unlike CPA, *C. perfringens* beta toxin (CPB) is a pore-forming toxin with a capsid, rim, and stem domain and produced by *C. perfringens* types B and C (Figure 4B) [55]. The CPB toxin disrupts the integrity of cell membranes by altering intracellular backbone proteins, signaling, and triggering inflammatory responses, among other pathways that lead to damage and inflammation in intestinal epithelial cells. Notably, endothelial cells and platelets are the target cells for CPB, and the CD31 protein, expressed explicitly on endothelial cells and platelets, functions as a CPB receptor (Figure 4A,B) [56,57]. CD31-deficient C57BL/6 mice demonstrated resistance to CPB [58]. CPB interaction with endothelial cells causes intestinal vascular necrosis and massive hemorrhage in the lamina propria and submucosa, primarily as small intestinal epithelial hemorrhage and necrosis [59]. CPB inhibits primary hemostasis by binding to the platelet plasma membrane, inhibits platelet aggregation, and impairs the ability of platelets to be activated by thrombin. (Figure 4A) [60]. Furthermore, the ability of CPB to bind to endothelial cells causes endothelial cell damage, increased vascular permeability and inflammatory response, localized tissue damage, as well as the exposure of subendothelial tissues and the initiation of endogenous coagulation, resulting in sustained bleeding from damaged vessels.

#### 2.2.3. Epsilon Toxin

Epsilon toxin (ETX) is a seven-pore toxin produced by *C. perfringens* type B that causes cell membrane permeability, resulting in cell lysis and death (Figure 4A). Proteolytic enzymes convert the initially less active 32.9 kDa prototoxin (P-ETX) to the 27 kDa active toxin within the intestine [61,62,63]. The ETX specifically targets lymphocytes, blood cells, and oligodendrocytes. ETX preferentially binds to lymphocytes with high levels of myelin and lymphocyte protein (MAL) and causes cell death, with the degree of this binding determined by the duration and concentration of ETX exposure [64,65]. ETX-mediated hemolysis in human erythrocytes is intricately linked to MAL and P2 receptors, specifically P2Y13 and P2X7. Erythrocytes contract in response to ETX, which exposes phosphatidylserine to the erythrocyte membrane. This process is accompanied by increased erythrocyte sphingolipid content, increased intracellular concentrations of calcium, sodium, and chloride ions, and a decrease in potassium ion concentrations. The ensuing intra- and extracellular ionic imbalance promotes hemolysis [66,67,68].

#### 2.2.4. Iota Toxin

Iota toxin (CPI) is a bacterial toxin consisting of two components: Ia and Ib produced by *C. perfringens* type E. (Figure 4B) [69]. Ia is an ADP-ribosylase that catalyzes ADP-ribosylation within the cell, disrupting cellular protein synthesis and energy metabolism. This process impairs cellular protein synthesis and energy metabolism. On the other hand, Ib, a membrane-associated component, binds to the cell membrane, causing intracellular sphingolipid accumulation. This disrupts the integrity and function of the cell membrane. The synergistic action of these two molecules is required to initiate the toxic effects [70]. The lipolysis-stimulated lipoprotein receptor (LSR) is the cellular receptor for Ib. Ib binds to amino acids 10–15 at the N-terminus of the LSR to generate the heptameric Ib, which subsequently binds to Ia (Figure 4B). As a result, the heptameric Ib binds to Ia, consequently increasing the inward flow of calcium ions and triggering the release of lysosomal proteases such as acid sphingomyelinase and histolytic protease. Acid sphingomyelinase hydrolyzes sphingolipids from the cell membrane, converting them into choline phosphate and ceramides, thereby promoting rapid endocytosis of CPIs [69,71,72].

#### 2.2.5. Enterotoxins and Necrotizing Enterocolitis B-Like Toxins

*C. perfringens enterotoxin* (CPE) consists primarily of an NH2-terminal region and a C-terminal structural domain (cCPE), which is formed only during spore production and is produced by *C. perfringens* F [73]. The toxin interacts with claudin receptors (such as Claudin-4) on the surface of intestinal epithelial cells. The binding of the c-CPE region of CPE to the receptor on the surface of intestinal epithelial cells induces dissociation of the tight junctions between cells. This process widens the gaps between the layers of epithelial cells, increasing the permeability of the epithelial cell barrier. As a result, molecules can readily flow through the cell gaps, compromising the integrity of the epithelial cell barrier [74,75,76,77]. The N-terminal structural domains reassemble, forming cation-selective β-barrel pores. These pores allow ions such as Ca^2+^ and small molecules to traverse the cell membrane. This process impairs cell membrane integrity, resulting in damage and, ultimately, death of intestinal epithelial cells [78,79,80].

Necrotizing Enterocolitis B-like toxin (NetB) is a 33 kDa pore-forming toxin. It interacts with cholesterol in cell membranes on the surface of susceptible cells, leading to the formation of pores of approximately 1.6–1.8 nm in diameter. The formation of these pores disrupts the cell membrane, causing an increased influx of calcium ions into the cell. This imbalance in intra- and extracellular ion homeostasis subsequently activates the apoptotic pathway in the host cell (Figure 4B) [81,82,83]. The precise pathogenic mechanism of the NetB toxin is currently being investigated.

## 3. Prevention and Treatment of *C. perfringens*

### 3.1. Vaccines

Recombinant vaccines for *C. perfringens* are used in animals, including pigs, cattle, sheep, goats, and horses. Additionally, oral bait pellet vaccines expressing toxins CPA, ETX, CPB1, and CPB2 have been developed using recombinant *Lactobacillus lactis* [84,85]. In countries such as the United Kingdom, Canada and China, appropriate vaccines have been developed and relevant property rights registered for the prevention of *C. perfringens* (Table 3). Most *C. perfringens* vaccines available on the market today are inactivated, subunit, and live attenuated. However, these vaccines have significant drawbacks. These include the need for multiple injections, specificity to particular toxins, and the lack of vaccines targeting enteric diseases [86]. To address these challenges, the integration of bioinformatics analysis, proteomics, and vaccinology can be employed to design more effective multi-epitope vaccines for the future development of *C. perfringens* vaccines [87,88]. Moreover, phage display technology can be used to produce phage display vaccines on a large scale within a short period and at relatively low cost. It can also facilitate the development of more efficient adjuvants. A path towards a more comprehensive, safer, and effective *C. perfringens* vaccine may necessitate the investigation of diverse vaccine routes, fostering interdisciplinary collaborations.

### 3.2. Antibiotics

Since the discovery of penicillin by Fleming in 1928, antibiotics, including azithromycin, ampicillin, penicillin, florfenicol, and metronidazole, have been widely employed for treating *C. perfringens* infections. However, the antibiotic resistance genes of *C. perfringens* have undergone horizontal transfer (transformation, transduction, conjugation) and vertical transmission in natural environments, *C. perfringens* is now resistant to ampicillin, streptomycin, tetracycline, penicillin, antimicrobial peptides, and other antibiotics [89,90]. Studies have shown that the transfer of clostridial plasmids (Tcp) plasmid in *C. perfringens* encodes antibiotic resistance genes, including chloramphenicol, clindamycin, erythromycin, bacitracin and lincomycin [21]. In addition, the tetracycline resistance genes, tetA(P) and tetB(P), encode proteins that mediate the mechanisms of active tetracycline efflux and ribosome-protected tetracycline resistance, respectively [91]. Target site modification, efflux pumps and drug inactivation confer resistance to macrolides. The erm gene of *C. perfringens* encodes a 23S rRNA methylase that mediates target site modification and confers resistance to macrolides. The highest antibiotic resistance rate of *C. perfringens* isolated from chicken and pork sold in Vietnam was tetracycline (91.30%), followed by clindamycin (43.48%) [92]. Whereas in China, *C. perfringens* resistance to clindamycin was as high as 49.66% in samples isolated from human sources [16]. Antibiotic resistance is a significant global health concern, accounting for approximately1.27 million (2019) deaths. The cumulative economic cost of antibiotic resistance is estimated at $a trillion, and the number of deaths is projected to increase to 20.3 million by 2024 [15,93]. Hence, given the resistance of *C. perfringens* to multiple antibiotics, there is a critical need for research and development into alternative therapeutic strategies. Exploring avenues such as microecological agents, herbal products, phages, and other innovative approaches has become imperative. Furthermore, by diversifying therapeutic modalities, there is a potential to reduce reliance on conventional antibiotics, lowering the risk of resistance transmission.

### 3.3. Probiotics

Probiotics in microecological preparations modulate intestinal microecological balance by increasing lipid metabolism, competitive inhibition, production of antimicrobial compounds, and stimulation of host immune system development, thereby protecting the host from *C. perfringens* invasion [94]. Some probiotics (i.e., *Bacillus subtilis*, *Lactobacillus*, *Bacillus velezensis* and *Bacillus polymyxa*) have been proven to prevent *C. perfringens* [95,96,97]. *Lactobacillus*, for example, produces antimicrobial compounds, such as bacteriocins. These compounds induce bacterial rupture and disrupt bacterial cell wall synthesis by increasing the permeability of the bacterial inner membrane [98]. *Bacillus amyloliquefaciens* also improves the gut microbiota following infection with *C. perfringens* [99].

### 3.4. Alternative Treatment

Plant extracts, herbal products, phage therapy, and fecal flora transplantation effectively treat *C. perfringens* infections [100]. Phytic acid has been demonstrated to inhibit the germination and growth of *C. perfringens* spores, making it a promising alternative food preservative for reducing *C. perfringens* contamination in various food products [101]. Arginine significantly attenuates α toxin-induced reduction in serum immunoglobulin IgA and IgG levels and intestinal morphological damage [102]. Organic acids can modify intestinal pH and other environmental factors, keep normal intestinal flora in balance, and alter the intestinal microenvironment, lowering the prevalence of *C. perfringens* infections [103]. Furthermore, yam was found to alleviate ampicillin-induced antibiotic-associated diarrhea by increasing the levels of the probiotics *Bifidobacterium bifidum* and *Lactobacillus lactis* and promoting the restoration of bacterial community diversity [4]. Phage therapy is now widely used to manage multidrug-resistant bacteria, and *C. perfringens*-specific phages have been identifieds [104]. Moreover, fecal flora transplantation involves introducing the gut microbiota from a healthy donor into the gut of a patient to restore balanced and normal gut flora. Fecal flora transplantation has been investigated as a potential treatment for *Clostridium difficile* infections [105].

## 4. Conclusions and Prospects

*C. perfringens* is a common member of the human gut microbiota, but its role as a harmless commensal or a potential pathogen remains controversial. Under normal conditions, *C. perfringens* maintains intestinal homeostasis together with other microorganisms. However, when the host immune function is reduced, the intestinal flora is dysbiotic, or when broad-spectrum antibiotics are used, *C. perfringens* proliferates and produces toxins, leading to enterotoxemia, ADD, and food poisoning. Therefore, *C. perfringens* may be either a harmless bystander in the gut or a serious threat under specific conditions, and further study of its behavioral mechanisms is essential for the prevention and control of related diseases. Based on the principle of “one world, one health”, managing this pathogen and promoting the investigation in this field warrants the following: (1) Explore in-depth the pathogenic mechanism. *C. perfringens* can produce various toxins and enzymes, but only a subset has been examined, leaving a lack of comprehensive understanding. Utilizing high-throughput sequencing, multi-omics approaches, and microbial culture technologies for in-depth research on the pathogenic mechanism of *C. perfringens* exotoxins and enzymes will establish a more robust scientific foundation for treating *C. perfringens*-related diseases. (2) Reducing the spread and infection of *C. perfringens*. Implement biosecurity measures to boost immunity and actively develop novel vaccines. (3) Implement personalized precision medicine. Developing multi-faceted and multi-pathway therapy is critical for precisely managing *C. perfringens* infections and reducing antibiotic resistance. This involves exploring resistance-reducing alternatives such as microecological agents, phage therapy, and bacterial colony transplantation.

## Figures and Tables

**Table 1:** Prevalence Rate and Number of Infections of *C. perfringens* in Humans.

Site	Disease	Prevalence Rate (%) or Number of Infections	Reference
USA	Foodborne illness	107	[19]
Canada	Foodborne infection	1.6 × 105	[20]
England	Foodborne illness	9 × 106	[21]
Iran	Antibiotic-associated diarrhea	15%	[30]
—	Antibiotic-associated diarrhea	5%–20%	[16]

**Table 2:** Prevalence Rate and Number of Infections of *C. perfringens* in Animals.

Year	Site	Disease	Species	Prevalence Rate (%) or Number of Infections	Reference
2013–2016	Switzerland	—	Horse	8%	[35]
2014–2015	Samsun (Turkey)	Septicemia	Sheep	45.2%	[36]
2021–2022	Kalubiya and Menofia (Egypt)	—	Lamb	17.5%	[37]
2000–2021	India	—	Pig	60%	[38]
			Sheep	56.3%	[38]
			Goat	38.7%	[38]
			Cattle	35%	[38]
—	Weifang, Guangdong, Taian, Pingying (China)	—	Chicken	38.42%	[36]
2019–2020	Gansu (China)	—	Sheep	14.7%	[39]
2020	Anhui, Guangdong, Guangxi, Fujian (China)	—	Ruminant	44.173%	[40]

**Table 3:** *C. perfringens* vaccines.

Year	Nation	Type of Vaccine	Species	Advantages	Patent Number
2018	China	Subunit vaccine	—	Simple preparation process, low immunizing dose, higher vaccine efficacy	CN107753940A
2017	China	Toxin vaccine	Cattle and Sheep	Strong immunity, and no toxic or side effects	CN107875377A
2013	South Korea	Genetically engineered vaccine	Poultry	Safe and efficient	KP101293667B1
2008	Canada	Peptide vaccine	Poultry	Safe and cost-effective	CA2685533A1
2008	China	Attenuated live vaccine	—	Safe and strong immunity	EP2007420A2
1995	UK	Genetically engineered vaccine	—	Safe and easy to produce	EP0642581A1
